# Evaluation of the Most Current and Effective Methods in the Analysis of Chlorinated Dioxins in Ground Beef

**DOI:** 10.1100/tsw.2003.87

**Published:** 2003-09-23

**Authors:** Ebere C. Anyanwu, Mohamed H. El-Saeid, Akpan I. Akpan, Mahmoud A. Saled

**Affiliations:** Department of Chemistry, 3100 Cleburne Street, Texas Southern University, Houston, TX, USA

**Keywords:** chlorinated dioxin, ground beef contamination, pesticides, analysis, United States

## Abstract

Chlorinated dioxins are the group of environmental pollutants consisting of 210 chlorinated dibenzo-p-dioxins and dibenzofurans. They are highly toxic and persistent. They are lipophilic and can easily biomagnify in the food chain, hence posing a serious threat to human health. The daily consumption of low-level contaminated food, mainly of animal origin, leads to the accumulation of dioxins in the human body. The exposures of the general human population to dioxins and the specific issues of a risk assessment of dioxin pose serious concerns in public environmental and nutritional health. This paper reviews the analysis of chlorinated dioxins in ground beef. The sources of contamination of chlorinated dioxins in ground beef are first reviewed to form a basis for a clear understanding of the health implications of chlorinated dioxins in the human food chain and why it is necessary to monitor the level of dioxins in animal food products, especially ground beef. The methods of collection, sampling, and processing of ground beef, and the methods of sample clean up prior to the analysis, are reviewed. Emphasis is laid on the new techniques that are available and that might be effective in the analysis of chlorinated dioxins in ground beef. Among these new methods and techniques are: the synergistic combination of ELISA/GC/MS, direct sample introduction to /GC/MS-MS, automated clean-up method, and the supercritical fluid extraction methods. The possible treatments of results from each method and technique are discussed and their respective efficiencies are compared. Finally, quality control and quality assurance parameters are evaluated for levels of accuracy, reproducibility, and precision.

## INTRODUCTION

Dioxins are the common name for the group of environmental pollutants consisting of 210 chlorinated dibenzo-p-dioxins (PCDD) and dibenzofurans (PCDF). They are highly toxic and persistent, especially if they are substituted with chlorine in the 2,3,7,8 positions. Furthermore, they are lipophilic and biomagnify in the food chain, hence posing a serious threat to human health[1,2,3,4]. They appear to act by binding to the Ah (aromatic hydrocarbon) receptor and to produce a broad range of adverse effects including, but not limited to, enhanced tumorigenicity, enzyme induction, immune suppression, and a wasting syndrome. In 1994, the U.S. Environmental Protection Agency (EPA) issued a draft risk assessment on dioxins and it indicated that >90% of dioxin exposure in the U.S. came from the diet, primarily from consumption of animal products. The EPA and U.S. Department of Agriculture (USDA) then conducted joint national surveys of dioxins in beef, pork, and poultry to obtain background levels in these products and the primary source of contamination in animal and human food. The evidence gathered thus far suggests the primary source of dioxin contamination in food animals is from their diet and that the nonfodder components of the diet deserve additional scrutiny. The congeners are detected in beef and the most frequently detected in beef are 1,2,3,4,6,7,8-HpCDD and OCDD[2,5,6,7].

Chlorinated dioxins are usually produced mainly during combustion processes in the presence of a chlorine donor and as by-products of the chlorine-processing chemical industries[8]. Chlorinated dioxins are persistent and ubiquitous in the environment and enter the human food chain through dioxin-contaminated animal products containing fats (i.e., meat, dairy, and eggs) that are ultimately consumed by humans. Because of their persistence and accumulation in the environment, their analysis has become part of monitoring programs in environmental and nutritional toxicology. Chlorinated dioxins are lipophilic in behavior and it is this quality that influences their accumulation in body fat and prevents them from being excreted in the urine. Several dioxins, particularly 2,3,7,8-tetrachlorodibenzo-p-dioxin (TCDD), are extremely toxic and stable compounds and they persist for years in the environment. They are able to induce numerous clinical conditions in animals such as carcinogenicity of dioxins, dysfunctions of the sexual and thyroid hormone systems including endometriosis, decreased spermatogenesis, hypothyroidism, and disturbed psychomotor development in the offspring[9]. However, in humans a series of conditions such as hormonal dysfunction, undescended testis, decreased spermatogenesis, testicular cancer, and endometriosis have been associated with chronic dioxins exposures[10].

## AIM OF THIS REVIEW

The aim of this review paper is to give an overview of the analysis of chlorinated dioxins in ground beef; the methods of collection, sampling, and processing of ground beef; the methods of sample clean up prior to the analysis; and the quality control and assurance parameters.

### Sources of Contamination of Chlorinated Dioxins in Ground Beef

The daily consumption of low-level contaminated food, mainly of animal origin, leads to the accumulation of dioxins in the human body[11]. The exposures of the general human population to dioxins and the specific issues of a risk assessment of dioxin exposures pose serious concerns in public environmental and nutritional health.

The most important route for human exposure to chlorinated dioxins is food consumption contributing over 90% of total exposure, with products of animal origin and fish making the greatest contribution to this exposure[12]. The dietary intake of dioxins in industrialized countries is on average 1–3 pg of TEQ (toxicity equivalent quantity) per kilogram body weight per day, and this level could be about two to three times higher if the dioxine-like PCBs are taken into consideration[12]. In the U.S.

especially, the concentration of dioxins in adults is 42% of dietary TEQ intake[13]. The individual adipose tissue dioxin contamination in nonoccupationally exposed persons living in France, in relation to the corresponding individually evaluated mean daily dietary dioxin intake, showed adipose tissue polychlorinated dioxins levels of between 18.5 and 76.9 pg I-TEQ/g lipids (mean value: 35.6 +/– 14.8), and these levels are similar to levels in other European countries and the U.S. without relation to sex or age[9]. Geographical surveys of domestic beef indicate that when beef samples are contaminated with significant levels of dioxins, it is due to pentachlorophenol-treated wood in animal feeding facilities. The absorption and distribution of dioxins in beef cattle can be determined through a controlled feeding study and related metabolism studies in calves and model animals.

Byard[14] reviewed the reports of PCDDs and PCDFs in human tissues to assess their toxicological significance and found that the predominance of 2,3,7,8-chlorinated congeners in human tissues and in the food chain suggested that the food chain was the major source of human dioxin residues[14,15]. Congener-specific analyses of seven PCDDs performed on 197 foodstuffs samples of animal origin from Belgium during years 2000 and 2001 found a relative contribution of 2,3,7,8-TCDD, 2,3,7,8-TCDF, 1,2,3,7,8-PeCDD, and 2,3,4,7,8-PeCDF to the PCDD/Fs TEQ of 85 +/– 7.9% mainly beef, accounting for roughly one-third of all food intake. A surveillance program on chlorinated dioxins in 29 foodstuff samples produced all over the four provinces in Catalonia (Spain) found the total TEQ values above that proposed by the World Health Organization (WHO) for beef[10,15,16,17,18,19]. Pentachlorophenol-treated wood components in the pole barn where the feeding experiment was conducted were found to have contributed to the animals' exposure[20].

In many countries, the total daily intake is of the same level, close to 2 pg TEQ/kg BW/day and the highest concentrations of chlorinated dioxins have been measured in beef. It was found that adults, neonates, and probably older children have a higher exposure level. If human exposure to chlorinated dioxins is higher than the most common tolerable daily intake (TDI) of 10 pg TEQ/kg BW/day, effective means to control the exposure level are needed[19,20,21,22].

## METHODS OF DETERMINATION

### Sample Collection

Ground beef (with % fat) samples can be obtained from three to five different food stores. Dry Florisil powder, 100–200 mesh (Fisher Scientific, Fair Lawn, NJ) with no furnace reactivation is used as received. All the samples should be beef only and their lipid contents should be specified. This would help in determining the levels of polychlorinated dioxins in ground beef in pgTEQ/g wet weight, respectively. The ground beef samples should then be prepared with three nominal levels of fat content containing approximately 10, 20, and 30% fat. Beef trimmings are utilized and initially ground through a 13-mm plate and then mixed in a ribbon mixer for additional homogeneity. This substrate is then reground through a 3-mm plate, to yield a consistency typical of commercial ground hamburger, and then further homogenized in bowl cutter assembly to yield a very fine sample of paste-like consistency, which appears homogeneous to the eye, lean and fat particles being indistinguishable.

All samples should be collected within the same day using appropriate sampling package and processes. The sampling package will contain the following items: two dioxin-free 500-ml wide-mouth amber glass jars with Teflon-lined polyurethane lids. One jar is for the sample. Every effort should be made to identify integrated animal renderers (packer renderer associated with a slaughter facility) when collecting the beef fat samples. To avoid contamination of the sample, the container should be kept covered and opened only when the sample is about to be processed in the laboratory. When collecting the sample, gloves and the above-mentioned glass jars should be used. If the samples are collected from more than one sample source, each container with the correct sample zip-lock bag should be labeled, and bubble wrap and flexible coolant packs should be used to protect the samples in transit. The samples should be maintained in the dark and under freezing conditions after collection until use in the lab.

## Sample Clean Up and Analysis

Extraction procedures will vary depending on the specific matrix but will generally involve solvent extraction followed by the "clean-up" and analytical procedures[1]. After extraction, extracts are cleaned up by sulfuric acid impregnated silica gel, purified on a series of silica gel, alumina, carbon column chromatography and then analyzed by high-resolution gas chromatography/high resolution mass spectrometry[23]. Or, for the clean-up columns, 5 g of Florisil is added to 10-mm i.d. × 330-mm length glass chromatography columns, each containing a sealed-in coarse fritted disk (Kontes, Vineland, NJ). The packed columns are then washed with 25 ml of acetonitril before use. Vacuum suction is used to speed up chromatography, but the columns are not permitted to become dry before use. Current methods for the extraction of fats from meat samples generally require the use of a relatively large quantity of organic solvent, such as hexane or ether. Since the use of organic solvents has some adverse environmental impact, and at the same time has high costs of purchase and disposal, the EPA has directed government agencies to reduce consumption of solvents in Federal laboratories[24].

New analytical methods are needed to expand the range of chlorinated organic analytes that can be detected in ground beef in a more efficient process. These new analytical methods include the use of modern methods and approaches, which are capable of evaluating, screening, quantifying, and/or confirming the presence of chlorinated dioxin residues. Most popular of these advanced technologies include hyphenated gas (GC) or liquid (LC) chromatography/mass spectrometry (MS) (or tandem mass spectrometry - $MS^n$), supercritical fluid extraction (SFE), aqueous microdialysis, solid phase extraction (SPE), and other techniques. By providing better methods for risk assessment, increasing laboratory productivity, and potentially reducing the amount of chemical residues in animal meat products, the methods and techniques help improve and maintain a safe beef supply.

## Synergistic Combination of ELISA/GC/MS

A reasonable separate number of test samples will initially be tested by enzyme-linked immunosorbent assay (ELISA) and by gas chromatography/mass spectrometry (GC/MS) to assess whether the ELISA performed on samples might be reliable enough for identifying a dioxin contamination in the ground beef. In addition, it would be beneficial to develop a method for evaluating the level of dioxin toxicity in humans who consume the ground beef by ELISA[25]. In this instance, one would expect the concept of TEQ screening could be used to validate the outcomes by comparing TEQ values for a set of ground beef samples to the ELISA responses predicted for those samples. It is prudent enough to expect a fairly good correlation between immunoassay and GC/MS for the ground beef and waste products from humans who ate them. This synergistic combination of ELISA/GC/MS should be useful for biological samples monitoring.

## Direct Sample Introduction to /GC/MS-MS

Currently, the analytical method used for persistent organic residues in animal food products is very laborious and time-consuming. Direct sample introduction to /GC/MS-MS in the monitoring of the chlorinated dioxin residues requires that a rapid, sensitive, quantitative, confirmatory, simple, inexpensive, safe, and rugged procedure has to be developed to monitor chlorinated dioxin levels as low as 1 ng/g. This approach avoids the costly, time-consuming, and labor-intensive clean-up and solvent evaporation steps associated with traditional methods and also has the potential to make a strong impact in the analysis of many types of chlorinated dioxin and other semi-volatile chemicals in a variety of matrices, including ground beef. These techniques and methods may free the analysts of the need to use copious amounts of environmentally harmful solvents and reduced the time and cost of performing such assays. A recent major emphasis of the analytical research and development effort should be focused on the integration of the sample clean-up step into the analysis procedure. As opposed to solely using SFE with carbon dioxide as an extraction solvent, pressurized water extraction should be utilized in tandem with the above assisting techniques plus matrix solid phase dispersion in sample preparation prior to extraction.

## Automated Clean-Up Method

Gas chromatography with high-resolution mass spectrometry (GC-HRMS) is used for the chlorinated dioxins and dioxin-like fractions[4]. The analyses are performed by GC-HRMS at ppt level ($10^{-12}$ g/g sample). This is a simple automated clean-up method for chlorinated dioxins that include several types of chlorinated dioxins — including all congeners with a WHO toxic equivalent factor (TEF) — in a single extraction and single purification step[4]. This method uses the principles of chromatographic behavior of chlorinated dioxins on a variety of adsorbents, including basic alumina. It enables the partitioning of analytes through multicolumn sequences. Hence, it is useful in that it is appropriate for the differences in structural and electronic properties of the chlorinated dioxins. Also, the fractionation process available with the Power-Prep automated clean-up system enables rapid independent analysis of the different groups of compounds.

## Supercritical Fluid Extraction

Consequently, analytical SFE is an alternative to solvent-based extraction methods. SFE, utilizing supercritical carbon dioxide (SC-$CO_2$), is gaining acceptance due to carbon dioxide's relatively low toxicity and reasonable cost. Further, SFE methods employing SC-$CO_2$ are environmentally safe, resulting in reduced extraction times and automation with commercial instrumentation[25,26].

Extensive preliminary SFE studies on ground beef samples performed with commercial SFE instruments showed a 42% increase in the fat content of the sample using gravimetric techniques[25,26]. SFE is shown to have many advantages, which include: minimization of the time, effort, and cost, and improvement in the quality of the results of the analyses. It also provides significant, more statistically valid and accurate results for risk assessment, more information to understand the effects and mechanisms, allows for better verification of organic food labeling, and finally, reduces the potential for food that has been deliberately or accidentally adulterated by toxic chlorinated dioxins to reach the consumer.

## Quality Control/Assurance Parameters

Samples should be maintained at a freezing temperature in the dark prior to and after analysis to avoid photochemical depletion of the dioxins. Samples will be prepared and analyzed using methods and quality assurance/quality control procedures that generally follow those described by the EPA. The quality control measures help to evaluate the robustness of the new strategy compared with a reference method. Proper care is taken to minimize random errors during the processing, measuring of volumes, temperature taking, and timing. Precision (or reproducibility) will be determined by the measurements and analyses of duplicates of samples under the same conditions and within batches. Systematic errors (sampling and sampling preparation, the analytical method, faulty instrumentation, operational mistakes) will be avoided to minimize bias[27]. In addition, to avoid these systematic errors, the WHO/EPA recommended safety concentration limit should be considered in the analysis. The instruments will be operated well above the detection limit. The final concentration values for all test samples will be derived by taking into account the concentration of the analytes in the control samples and deducting the values from the overall concentration values.

## DISCUSSION

For the purposes of this report, it is advisable that the total whole weight TEQ for ground beef be calculated by assuming the lipid content of ground beef was 19% and by using one-half the detection limits to represent the concentration of nondetectable CDD/F congeners in the samples. Using this methodology, the total background TEQ is estimated in parts per trillion (ppt) for beef on a wet weight basis. Sample results are often expressed in parts per trillion (ppt) of toxicity equivalents, or TEQ. The TEQ concentration of individual congeners is calculated as the analytical concentration of that congener times its toxicity equivalency factor, or TEF. The TEF for TCDD and PeCDD has been set at 1 since they are the most toxic congeners. The TEFs for the other congeners are less than 1 and are based on their interaction with the Ah receptor compared to TCDD/PeCDD. The sum of the TEQ concentrations for the individual congeners is the TEQ concentration for the mixture. Since the TEQ concentration of a mixture of dioxin congeners is estimated to be equivalent to a concentration of just TCDD or just PeCDD, this provides consistency in addressing dioxin contaminations of various mixtures.

Between 1997 and 1999, several cases of dioxin contamination in foodstuffs of animal origin occurred in Europe due to feed contaminated by several independent sources. Investigation of a total of 128 samples of cow's milk, poultry, eggs, and meat revealed several cases of residue levels distinctly above the background exposure limit of approximately 2.5 ng of I-TEQ/kg (fat basis)[28]. The dioxins with no chlorines in the 4- and 6-positions were apparently metabolized, because residues were not detected in milk and body fat[29]. The question then is, what level of dioxins in ground beef would interfere with the human cellular metabolism, since meat products have been found to contain 0.52–9.08 pg WHO-TEQ/g fat with higher concentrations of dioxin contamination[10,30,31]? In the future, due to the results of these studies, the SFE methods could become more widely used in laboratories around the world that routinely monitor chemical residues in food.

## CONCLUSION

The analysis of chlorinated dioxins in ground beef has been reviewed. Although extraction procedures vary according to the specific matrix, generally, most methods involve solvent extraction followed by the "clean-up" and analytical procedures[1,32]. Nevertheless, all the methods require the minimization of the potential for contamination. It is of the utmost importance that one makes sure that the sampling occurs at renderer and processor sources (primary sites). The samples should be maintained at a freezing temperature in the dark prior to and after analysis to avoid photochemical depletion of the chlorinated dioxins. Samples should also be prepared and analyzed using methods and quality assurance control procedures that generally follow those described by the EPA. If the sample is to be collected at sites other than the ingredient manufacturer, it is important that the ingredient does not combine with any other ingredient(s) when collected. Of all the clean-up methods reviewed, the SFE method is highly recommended because it is a safe, rapid, selective, easy, and waste-minimizing approach to extraction, which in addition, uses pressurized and heated carbon dioxide to dissolve analytes of interest rather than liquid organic solvents.

## BIOSKETCHES

**Ebere C. Anyanwu** is a Clinical Neurophysiologist and Director of Cahers Neurosciences Research Inc., Houston, TX and also a member of the Biomonitor Research Group, Department of Chemistry, Texas Southern University, Houston. He is also an Adjunct Professor of Anatomy and Physiology at the North Harris and Montgomery Colleges in Houston and The Woodlands, respectively.

**Mohamed H. El-Saeid** is a Post Doctoral Research Associate and a member of the Biomonitor Research Group, Department of Chemistry, Texas Southern University, Houston. Mohamed El-Saeid was Assistant Professor of Food Chemistry in the Department of Food Science and Technology, Faculty of Agriculture at Al-Azhar University in Egypt.

**Akpan I. Akpan** is Associate Professor in the School of Public Affairs, Urban Planning and Environmental Policy and Management, Texas Southern University, Houston. Dr. Akpan was a professor of Urban Planning and Environmental Sciences in Nigeria for many years.

**Mahmoud A. Saleh** is Professor of Chemistry and the Director of the Biomonitor Research Group, Department of Chemistry, Texas Southern University, Houston. Professor Saleh was the Chairman of the Department of Chemistry and has supervised several Ph.D. students.
